# Elevated Telomeric Repeat-Containing RNA (TERRA) Levels Linked to Telomere Dysfunction and Telomerase Inactivity in Blood Cells of Children With Aplastic Anemia

**DOI:** 10.7759/cureus.71241

**Published:** 2024-10-11

**Authors:** Jayitri Mazumdar, Priyanka Chowdhury, Badal Chandra Mondal, Anjan Kumar Das, Utpal Ghosh

**Affiliations:** 1 Department of Pediatric Cardiology, Rabindranath Tagore International Institute of Cardiac Sciences, Kolkata, IND; 2 Department of Biochemistry and Biophysics, University of Kalyani, Kalyani, IND; 3 Department of Pediatrics, Calcutta National Medical College and Hospital, Kolkata, IND; 4 Department of Pathology, Calcutta National Medical College and Hospital, Kolkata, IND

**Keywords:** aplastic anemia, btbd12, parp-1, shelterin, telomerase, terra, trf1, trf2

## Abstract

Background

Aplastic anemia (AA) is characterized by pancytopenia and hypocellularity of the bone marrow. Certain inherited or genetic forms of AA have also been associated with telomere dysfunction. Here, we report the clinical manifestations of eleven AA patients aged between one and 12 years, along with the expression of a few candidate genes involved in the telomere length (TL) maintenance pathway.

Methods

The clinical manifestations were recorded for all the patients. The average telomere length of peripheral blood mononuclear cells (PBMC), the expression of telomerase subunits, telomere-associated proteins, and chromosome-specific telomeric repeat-containing RNA (TERRA) in whole blood cells of each patient was compared with an age-matched control group consisting of five clinically confirmed normal individuals.

Results

Out of 11 AA patients, four were found to have upper limb anomalies, and two showed short stature along with other defects. All the patients showed significantly shorter telomere length compared with the age-matched control group. The essential subunits of telomerase (hTERT and hTERC) were significantly low, and the shelterin protein is abnormally expressed in all patients implicating a compromised TL maintenance pathway. Notably, AA with combined androgen and prednisolone treatment showed a marked reduction of TERRA level than that of AA without androgen/prednisolone therapy.

Conclusion

Based on the findings and observations made, it appears that there might be an association between telomere dysfunction and elevated levels of TERRA in patients diagnosed with aplastic anemia who are 12 years of age or younger.

## Introduction

In India, about 55% of women, 24% of men, and 70% of children are found to be suffering from anemia [1]. Anemia is categorized into several types; the most common among them are iron deficiency anemia, thalassemia, sickle cell anemia, hemolytic anemia, pernicious anemia, acquired aplastic anemia, and congenital aplastic anemia. Here, we are concerned about aplastic anemia (AA), whose common pathogenic feature is a compromised telomere length maintenance pathway [2,3]. Aplastic anemia is an immune disorder that destroys hematopoietic stem cells and is heterogeneous in origin [4]. It may cause bone marrow failure, and not enough new blood cells are produced, leading to a serious, life-threatening situation [5]. Hematopoietic progenitor cells have limited proliferative capacity due to progressive shortening of telomere. So, their replicative capacity is restricted and ultimately leads to bone marrow failure. Congenital aplastic anemia, or Fanconi anemia, is a rare, X-linked, or autosomal recessive disorder characterized by bone marrow failure, congenital anomalies, and hypersensitivity to DNA interstrand crosslinks. Various studies have shown that leukocytes of patients with congenital and acquired aplastic anemia have short telomeres [2]. The telomere, at its critically short length, induces chromosome fusion, DNA damage-induced apoptosis, and growth arrest [2,3].

Telomere erosion can be reversed either by telomerase holoenzyme or by alternative lengthening of telomeres (ALT) where a large number of proteins are involved [6]. Human telomerase reverse transcriptase (hTERT) and telomerase RNA component (hTERC) are two essential subunits of the telomerase holoenzyme for its activity [7,8]. Several increasing reports show the mutation in the coding gene of hTERT and hTERC along with short telomeres in AA patients [9,10]. Most of these mutations result in reduced or no telomerase activity when cloned and overexpressed in telomerase-negative cells [11].

Six important ALT proteins called shelterin proteins, such as TRF1, TRF2, POT1, TPP1, TIN2, and RAP1, are key players in maintaining native telomere structure and length [12]. Day by day, new proteins are being discovered to be involved in telomere length maintenance, making this telomere metabolism more exciting. TRF2 can act as a protein hub to recruit other proteins that have a role in the ALT pathway. For example, BTBD12, a structure-specific endonuclease, is reported to interact with TRF2 [13]. The same TRF2 can be poly(ADP-ribosyl)ated by PARP-1, and uncapped telomere is protected [14]. MRN (MRE11/RAD50/NBS1) complex, especially NBS1, is required for the ALT mechanism [15]. MUS81 and BTBD12 can localize at telomere and are required for sister chromatid exchange (SCE) [16]. In fact, mutations of the genes NOP10 and NOLA1 and genes of shelterin proteins are also reported in aplastic anemia [17]. However, the proportion of hTERT and hTERC mutations is about 5-10%, whereas mutations in the genes of shelterin protein complexes are rare and mostly found in children [18]. So, in addition to gene mutations, other mechanisms must be involved in the shortening of the telomeres in aplastic anemia. Telomeric-repeat-containing RNA (TERRA) can be transcribed from the sub-telomeric to the telomeric region, and it can inhibit telomerase [19]. TERRA is also involved in telomere length maintenance and telomere integrity [20]. As such, there is no report of TERRA expression and its association with the disease anemia. So, there could be major three categories of molecules such as telomerase holoenzyme, telomere-associated proteins, including shelterin proteins, and TERRA that can modulate telomere length and telomere integrity.

Importantly, the hematological manifestations are not observed for congenital anemia below 14 years for most of the cases (~75%) [4,21] and early diagnosis is urgently needed in childhood to cure the patients. In this work, we present clinical manifestations for all patients. We compared the average telomere length of peripheral blood mononuclear cells (PBMC), expression of telomerase subunits, telomere-associated proteins, and TERRA of AA patients (one to 12 years) with the age-matched control group.

## Materials and methods

Control group and patients

Blood samples were collected from hospitalized (Calcutta National Medical College & Hospital, Kolkata, India) patients strictly following the guidelines of the Institutional Ethical Committee, Calcutta National Medical College (Ethical Approval No 95 dated 08.08.2013). We have eleven (n=11) clinically/pathology-confirmed AA patients with ages ranging from one to 12 years. For each patient, age-matched clinically confirmed five (n=5) normal individuals were treated as the control group. Each parameter of a patient was compared with the mean ± SD of the age-matched control group.

Stress test as detected by chromosome breakage

PBMC from both the patient and age-matched control group was treated with phytohemagglutinin (PHA) and cultured. After 24 hrs of incubation, the PBMC was treated with mitomycin C (MMC) and allowed to grow for 48 hrs further. Then the cells were processed for metaphase chromosome spreading following standard protocol for monitoring different chromosome aberrations.

Measurement of telomere length

Telomere length was measured by q-polymerase chain reaction (q-PCR) as described by Cawthon et al. [22]. The DNA was isolated from the PBMC using the protocol adapted from Santos et al. [23]. In brief, the DNA extraction was performed from PBMC using 500 μL STE solution (10 mL 1 M Tris-HCl, pH 8.0, 2 mL 0.5 M EDTA, pH 8.0, and 5 mL 3 M NaCl), 150 μL 20% SDS, and 20 μL proteinase K (5 mg/mL). Then it was incubated at 56°C for 18 h in a water bath, followed by the addition of 80% (v/v) isopropanol. Then it was centrifuged at 15,000 g for 30 min. Then the precipitated material was suspended in water, and the protein contamination was removed following the standard phenol-chloroform and isoamyl methods. Telomere length was measured using the primers as described by Cawthon et al. [22], and the PCR was done with isolated DNA as a template.

Estimation of gene expression of telomerase components and telomere-associated proteins by real-time PCR

Total RNA was isolated from the whole blood cells of the patient and a normal individual using an RNA isolation kit (Ambion Ribopure RNA isolation kit, Thermo Fisher Scientific Inc., Waltham, MA). The RNA (2 µg) was treated with DNase I (2 units/µl) for 30 minutes at room temperature, and the solution was heated at 70^o^C for 10 minutes to stop the reaction. Then it was chilled in ice, and reagents required for cDNA synthesis were added. The reaction was carried out at 37^o^C for one hour. Then it was heated at 95^o^C for five minutes, followed by incubation in ice for 10 minutes. This cDNA sample was used as a template for real-time PCR or stored at -20^o^C for subsequent experiments. Using Taqman primer, the expression of hTERT (Hs00972650_m1), hTERC (Hs03454202_s1), TRF2 (Hs00194619_m1), POT1 (Hs00209984_m1), TPP1 (Hs00166099_m1), TIN2 (Hs01554309_g1), PARP-1 (Hs00242302_m1), and BTBD12 (Hs00536164_m1) was monitored by real-time PCR (7900HT Fast Real-Time PCR System; Applied Biosystem, Life Technologies, Thermo Fisher Scientific Inc., Waltham, MA). We monitored the expression of TRF1 and RAP1 in real-time PCR using SYBR Green (Thermo Fisher Scientific Inc., Waltham, MA).

Expression of TERRA

Different human chromosomes have different telomere lengths, and hence the expression of TERRA from each chromosome is different. Here, chromosome-specific TERRA expression was done as per Porro et al. [24]. In brief, total RNA was isolated from whole blood cells using the TRIzol method. Complementary DNA (cDNA) prepared by reverse transcriptase as described above was used as the template for real-time PCR using SYBR Green (Thermo Fisher Scientific Inc., Waltham, MA).

Statistical analysis

Each experiment was repeated at least three times, and the mean ± SD of each patient parameter was compared with the mean ± SD of the same parameter of the respective age-matched control group using one-way ANOVA. The significance values (p-values) were written within brackets in the text.

## Results

Clinical results

Patients P1, P2, P3, P4, and P5 presented with pancytopenia (clinically manifested by pallor and bleeding manifestation), with the absence of hepatosplenomegaly, and bone marrow aspiration cytology confirmed the diagnosis. These five patients (mean age nine years) were treated with packed cells, platelet transfusion, and intravenous antibiotics. But all of them failed to show a good outcome. Two patients died due to severe infection and one due to massive hemorrhage within seven days of admission as they presented with severe pancytopenia (absolute neutrophil count (ANC) <500/mm3, platelet count <50,000/mm3). The remaining two patients improved with transfusion therapy.

**Figure 1:**
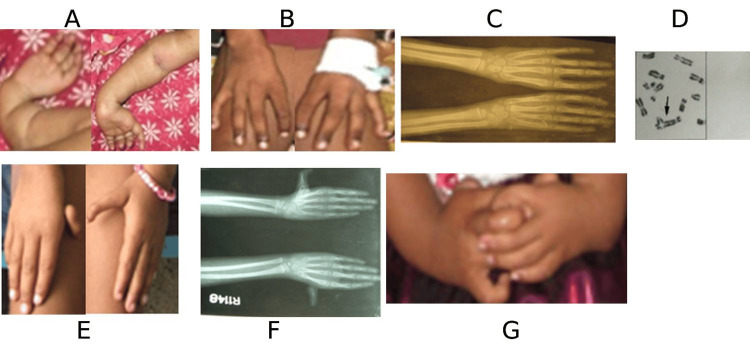
Physical abnormality and chromosomal aberration study A: cropped picture hands of patient P6 showing four digits with an absent thumb in both hands; B: cropped picture hands of patient P7 showing four digits with an absent thumb in both hands; C: an X-ray image of both hands of patient P7 showing absence of thumb in both hands; D: chromosomal aberration of patient P8; E: cropped picture hands of patient P10 showing anomaly in both thumbs; F: an X-ray image of both hands of patient P10 showing triphalangeal thumb in the left and absent first metacarpal in the right hand; G: cropped picture hands of patient P11 showing bilateral radial ray defect and absent radius on the left side

P6 was a one-year-old female patient with hypertelorism, stunting, fused kidney, and radial ray defect mainly on the right side with an absent thumb in both hands, having no hematological manifestation. The baby had no thumb, as shown in Figure 1A. P7 was a ten-year-old male child with four digits in both hands as shown in Figure 1B and the X-ray of hands in Figure 1C. He had right-sided dysplastic kidney, short stature, and mental retardation. This patient was treated with oral oxymetholone once-daily therapy after initial stabilization with transfusion therapy and showed a good response. Patients P6, P7, P8, P9, P10, and P11 showed positive stress tests as detected by significant (p≤0.001) chromosomal breakage after treatment with MMC compared with the normal group. The stress test showed a significant increase in the number of cells (~30%) having chromosome aberration after treatment with MMC compared with untreated cells (2%). P8 was an eight-year-old female having short stature, mental retardation, and hyperpigmentation of the body. No radial ray was detected, and no renal anomaly was detected by USG (data not shown). A typical picture of chromosomal aberration in patient P8 is shown in Figure 1D. This patient was initially stabilized with packed cell and platelet transfusion and discharged with oral androgen (tab stanozolol 4 mg twice daily). The patient showed a good response to stanozolol therapy, and to date, she is in three monthly follow-ups. P9 was a five-year-old female, the younger sister of P8, having the same features as P8, like short stature, mental retardation, and hyperpigmentation of the whole body. She presented with pancytopenia, and a bone marrow biopsy showed aplastic anemia. Unfortunately, this patient showed a poor response to oral stanozolol therapy and succumbed to death. P10 was a nine-year-old female with the anomaly in both thumbs as shown in Figure 1E. She had a right-sided thumb attached to the hand with a skin tag and a triphalangeal thumb on the left side, as shown in the X-ray of her hands in Figure 1F. Both the kidneys were normal. She had pancytopenia and a bone marrow biopsy revealed aplastic anemia with a positive chromosomal breakage study. She responded well to the combined therapy of oral stanozolol and prednisolone. Initially, prednisolone was given daily, and after counts showed a significant rise, prednisolone was tapered to alternate days (0.5 mg/kg/dose). P11 was a 1.5-year-old female child having a bilateral radial ray defect with an absent radius on the left, as shown in Figure 1G, and a bilateral absence of the first metacarpal with both the thumbs hanging by skin tags (X-ray not shown) and orbital mass on the right side. No hematological manifestation has been observed to date.

Stress test as detected by chromosome breakage study

Generally, Fanconi anemia patients lack repair of inter-strand crosslinked (ICL) DNA, and so they are very sensitive to interstrand crosslinking agents like MMC. We observed higher chromosomal breaks in MMC-treated PBMC for patients P6-P11 compared with that of normal individuals. Various types of chromosomal aberration were observed (data not shown).

Reduction of telomere length of all patients

**Figure 2:**
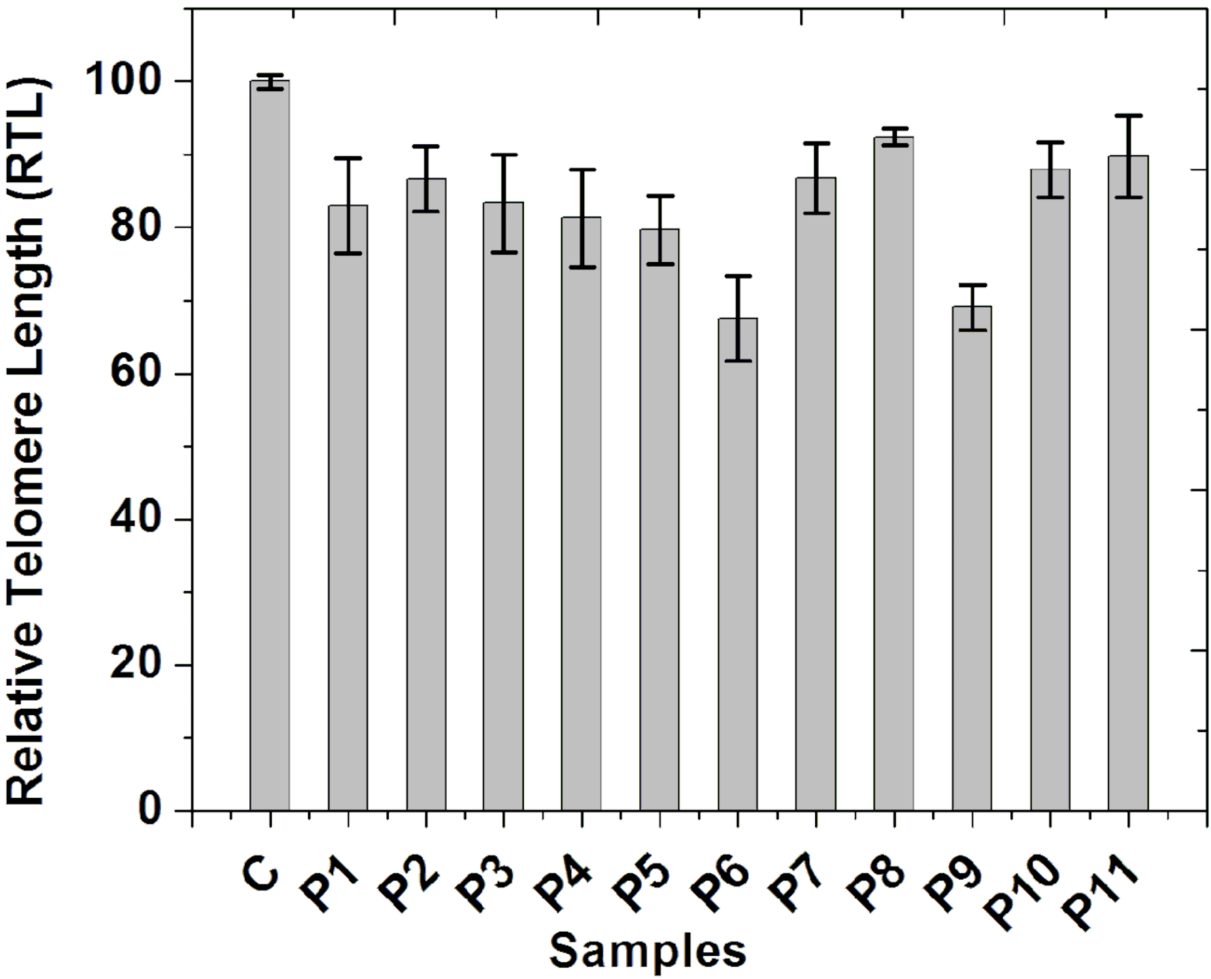
Telomere length (TL) measurement Relative telomere length (RTL) (%) of patients is shown here compared with that of the age-matched control group (C).

We measured the average telomere length of all the patients and the control groups and compared it. The telomere length of each control group was taken as 100%, and accordingly, the average telomere length of each patient was calculated, which varied from 67% to 92%. However, we observed that the average telomere length of all the patients was significantly (p≤0.05) shorter than the respective age-matched control group. The data is shown in Figure 2.

The shutdown of hTERT and hTERC: two essential subunits of telomerase

**Figure 3:**
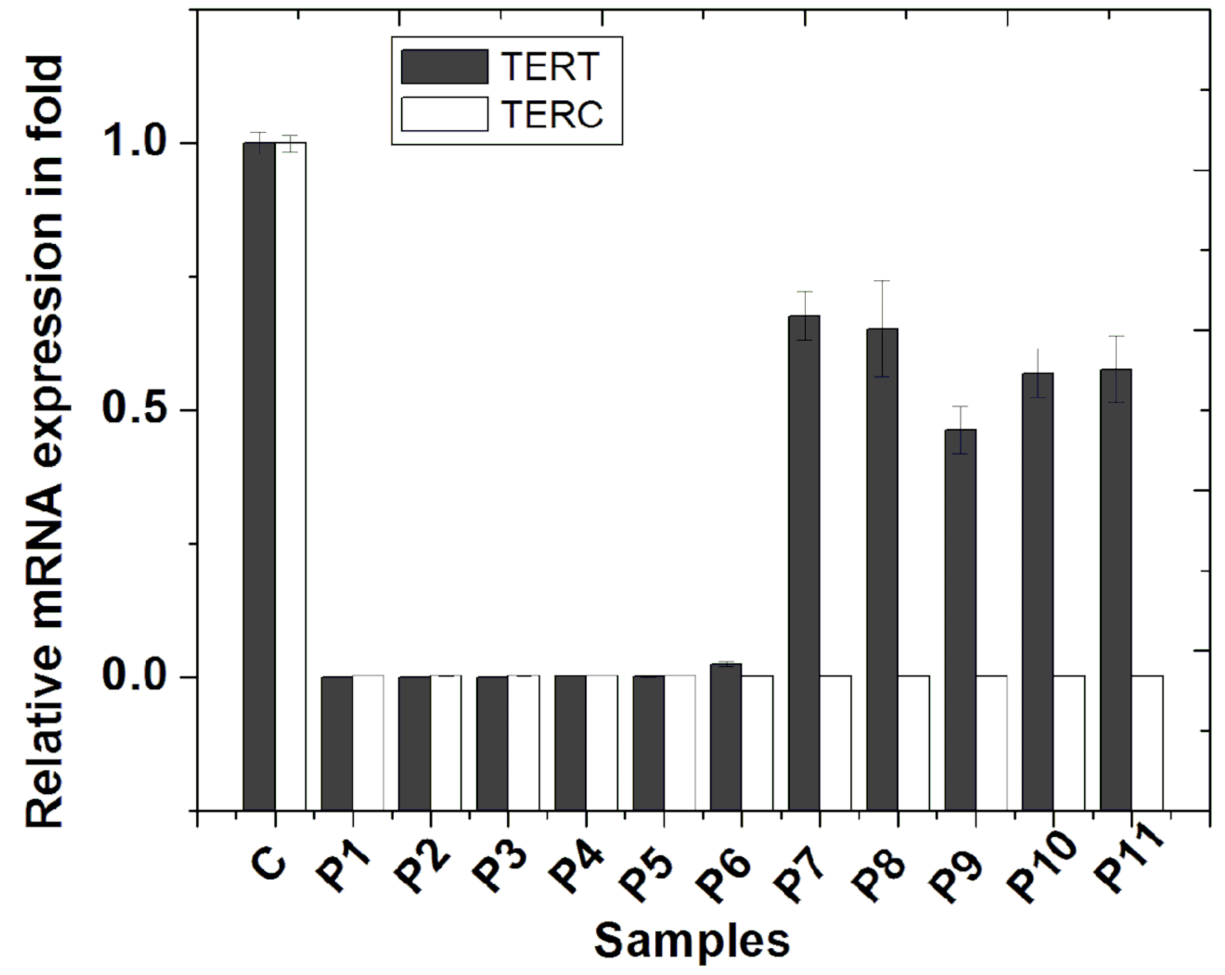
Relative mRNA expression of human TERT and TERC as detected by real-time PCR using the Taqman probe Expression of each subunit in the age-matched control group (C) is taken as 1, and accordingly, expression of each subunit in each patient is normalized as shown in the bar diagram. TERT: telomerase reverse transcriptase; TERC: telomerase RNA component; PCR: polymerase chain reaction

We checked the expression of hTERT and hTERC in all 11 patients and found a significant (p≤0.001) reduction of both genes concerning the age-matched control group as shown in Figure 3. The hTERT expression was negligibly small in P1-P6, whereas that in P7-P11 varied from 48% to 74% compared with normal. Notably, the expression of hTERC was reduced to below 1% in all the patients. These data indicate that all the patients have inactive telomerase in their whole blood cells.

Gene expression of a few telomere-associated proteins

**Figure 4:**
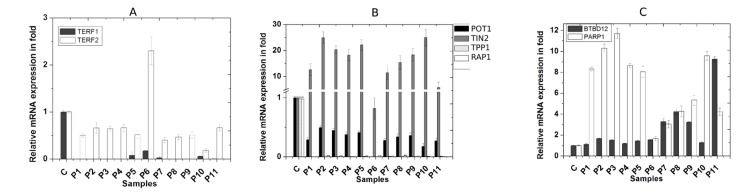
Relative mRNA expression of six shelterin and two non-shelterin telomere-associated proteins as detected by real-time polymerase chain reaction (PCR) Expression of each protein in the age-matched control group (C) is taken as 1, and accordingly, expression of the proteins in each patient is normalized. A: relative expression of TRF1 and TRF2; B: relative expression of POT1, TIN2, TPP1, and RAP1; C: relative expression of PARP-1 and BTBD12.

We observed the expression of all six shelterin proteins (TRF1, TRF2, TIN2, TPP1, POT1, and RAP1) and two non-shelterin proteins like PARP-1 and BTBD12 using real-time PCR, as shown in Figures 4A, 4B, 4C. All the shelterin proteins except TIN2 were reduced significantly in all these patients, as shown in Figures 4A, 4B. However, only P6 showed an increased level of TRF2. Notably, the TIN2 level was increased significantly in all the patients except P6, as shown in Figure 4B. This deregulated expression of shelterin proteins implicates loss of the native capping structure of telomere in all patients in their whole blood cells. Such up-capped telomere may lead to chromosomal fusion and induce DNA damage response pathways. PARP-1 is a well-known DNA repair protein and generally up-regulates after DNA damage. We observed significantly (p≤0.01) higher expression of PARP-1 compared with the control group as shown in Figure 4C, probably due to uncapped telomere, which may induce DNA damage response. We observed significant (p≤0.01) up-regulation of BTBD12 in all the patients (except P1, P4, and P10) with different extents, as shown in Figure 4C.

TERRA expression

**Figure 5:**
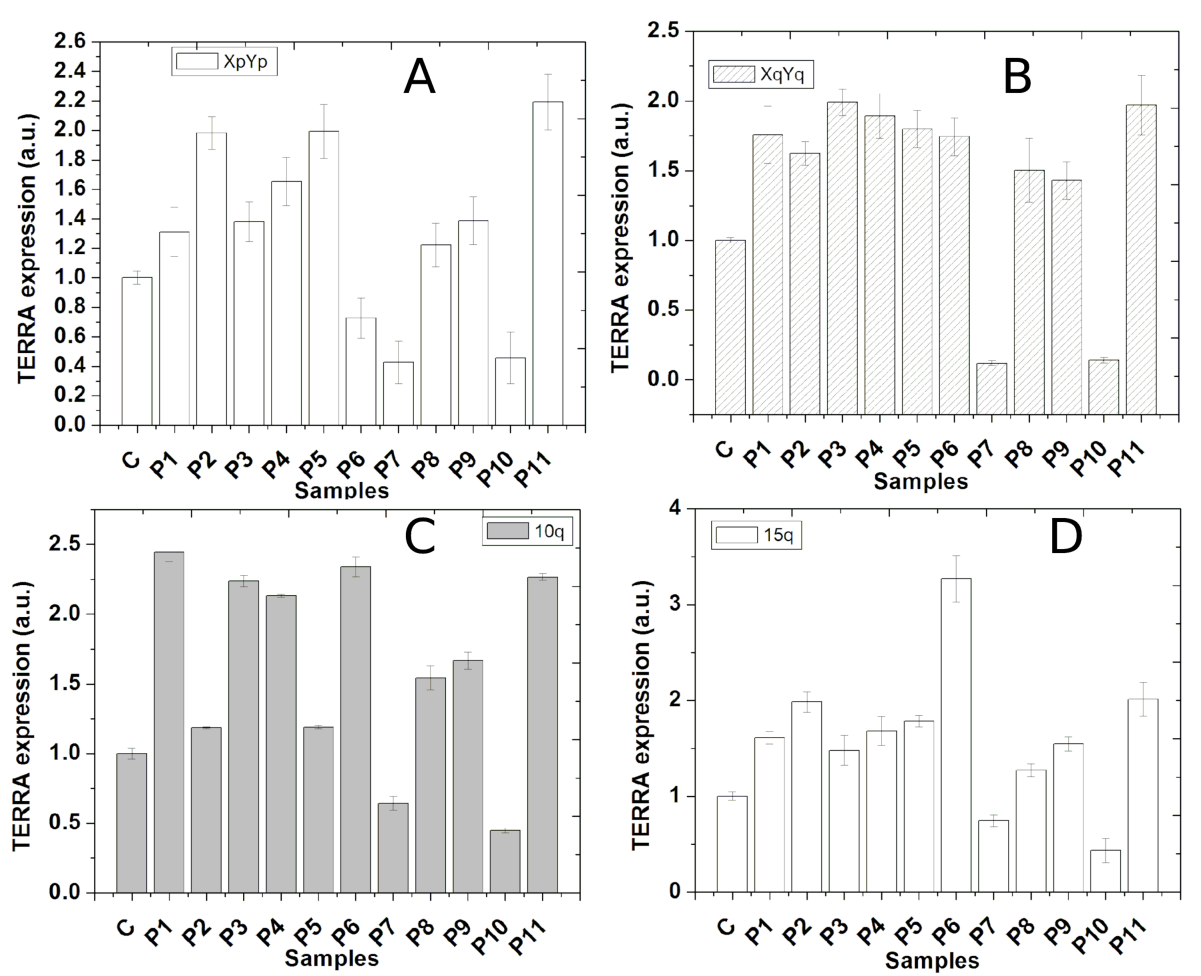
Chromosome-specific TERRA expression in the blood cells of patients in comparison with age-matched control group (C) Expression of TERRA in the control group is taken as 1, and accordingly, TERRA expression in patients is normalized. A, B, C, and D show TERRA levels from the chromosomes XpYp, XqYq, 10q, and 15q, respectively. TERRA: telomeric repeat-containing RNA

We checked chromosome-specific TERRA expression in all the patient samples and compared them with the age-matched normal control group as shown in Figure 5. All the patients except P7 and P10 showed significant (p≤0.01) higher TERRA levels for all four chromosomes compared with the respective age-matched control group. Notably, P7 and P10 patients showed consistently significant (p≤0.01) lower TERRA levels in all four chromosomes, as shown in Figures 5A, 5B, 5C, 5D. We observed that telomere damage or disruption of native telomere structure increased TERRA levels in cultured A549 cells [25]. Here also the TRF2 level was significantly lower in all patients (except P6), telomere-associated proteins were deregulated implicating dysfunctional telomere, and we observed a significantly higher TERRA level. So, all the patients have dysfunctional telomere, and that causes up-regulation of TERRA. However, four patients, P7, P8, P9, and P10, were treated with oral androgens with prednisolone, and two of them, P7 and P10, responded well to the treatment and showed low TERRA levels. P9 did not respond to the therapy and died and showed TERRA level high compared with P7/P10. P8 also responded to androgen therapy and showed a TERRA level intermediate between P9 and P7/P10. So, a high TERRA level is associated with aplastic anemia. Those who responded well against combined androgen with prednisolone therapy showed low TERRA levels. The list of primers used for TERRA expression is given in Table 1.

**Table 1:** List of primers used for TERRA expression using SYBR-Green q-RT PCR TERRA: telomeric repeat-containing RNA; RT PCR: real-time polymerase chain reaction

TERRA	Forward 5’-3’	Reverse 5’-3’
TERRA 10q	GAATCCTGCGCACCGAGAT	CTGCACTTGAACCCTGCAATAC
TERRA 15q	CAGCGAGATTCTCCCAAGCTAAG	AACCCTAACCACATGAGCAACG
TERRA XpYp	GCAAAGAGTGAAAGAACGAAGCTT	CCCTCTGAAAGTGGACCAATCA
TERRA XqYq	GGAAAGCAAAAGCCCCTCTGAATG	ACCCTCACCCTCACCCTAAGC
36B4u	CAGCAAGTGGGAAGGTGTAATCC	CCCATTCTATCATCAACGGGTACAA

## Discussion

We examined 11 patients with aplastic anemia without categorizing them into specific types. Despite this, our analysis revealed stress test positivity in patients P6-P11, suggesting a possible Fanconi anemia subtype. Genetic testing beyond this was not conducted. We described the clinical manifestations for all patients. Shortened telomeres were uniformly observed across all patients, albeit with some variation in length. Previous studies have linked aplastic anemia to telomeropathies [1-3]. Two well-known ways of replenishment of telomere length shortening are telomerase and the ALT pathway. Shelterin and non-shelterin telomere-associated proteins are involved in the ALT pathway [13]. So, it is expected that the patients will have either compromised telomerase activity or ALT pathway. Here, we observed inactive telomerase and de-regulated expression of candidate genes involved in ALT pathways in all patients. We observe a significant reduction of two essential subunits of telomerase, hTERT, and hTERC, implicating inactive telomerase. All shelterin and two TRF2-interacting non-shelterin proteins are highly deregulated, which possibly alters the ALT pathway in these patients. Several reports show mutation of hTERT and hTERC genes in aplastic anemia [9,10]. Reduction of hTERT or hTERC expression due to mutation in the regulatory part of the genes, such as in the promoter region, has been observed in human cancer or other telomere diseases [18]. So, a mutation in the promoter region of the gene hTERT/hTERC may reduce its expression. Furthermore, aplastic anemia or bone marrow failure patients have a variable sequence of hTERT with inactive telomerase activity [26]. However, we did not check such mutations. So, it would be worthwhile to check promoter mutations of telomerase subunits in aplastic anemia.

Shelterin proteins TRF1 and TRF2, crucial for maintaining telomere structure, were notably depleted in all patients, except P6 for TRF2. This depletion likely contributed to dysfunctional telomeres. Moreover, other shelterin proteins exhibited significant reductions compared to controls, with TIN2 showing notably elevated levels, except in P6.

Shelterin proteins TRF1 and TRF2 play a key role in stabilizing the capped structure of the telomere, and depletion of these proteins makes the telomere uncapped, which triggers a DNA damage response signal [12,27]. Here we see that TRF1 is significantly low in all patients and TRF2 is very low in all patients except P6. We observed that the knocking down of TRF1 and TRF2 makes the telomere uncapped, producing telomere-induced foci or dysfunctional telomeres in cultured A549 cell lines [25]. Depletion of TRF2 has also been reported to make the telomere uncapped or dysfunctional telomere [27,28]. So, these patients most likely have dysfunctional telomere. Other shelterin proteins TPP1, RAP1, and POT1 are also seen as significantly low in all patients compared with the age-matched control group. Notably, TIN2 is several folds (5 - 25) higher than the control group in all patients except P6. This data implies that a high level of TIN2 is most likely associated with this aplastic anemia. This aberrant expression pattern of shelterin proteins suggests a potential association with aplastic anemia, although further research is warranted for confirmation. This aberrant expression of shelterin in patients most likely interferes with ALT pathways for telomere length maintenance. Dysfunctional telomeres can induce chromosomal breakage, exacerbated by treatments such as MMC, as observed in stress tests of patients P6-P11. Generally, PARP-1 gets hyperactivated immediately after DNA damage. Here, we see PARP-1 up-regulation with different extents in different patients ranging from 1.5 to 12 fold, possibly due to DNA damage triggered by telomere dysfunction. However, most patients are associated with over-expression of BTBD12, a candidate for telomere recombination. We do not know the reason behind it. Possibly, telomere damage may trigger a homologous recombination pathway where BTBD12 is a key player. BTBD12 can interact with TRF2 and recruit other structure-specific endonucleases required in the recombination-based ALT pathway [13,16]. So, hyper-expression of BTBD12 in aplastic anemia could be an attempt to increase telomere recombination via the ALT pathway to replenish telomere erosion. DNA damage and TRF2 loss induce TERRA up-regulation in HeLa cells [29]. We also observed that telomere damage or depletion of TRF1/TRF2 increases TERRA levels in A549 cells [25].

Here, we see significantly high TERRA levels in all patients having short and dysfunctional telomeres. Four patients (P7, P8, P9, and P10) were given combined androgen and prednisolone therapy. Out of these four patients, two (P7 and P10) show good response and consistently show lower TERRA levels for all four chromosomes. P9 did not respond to such therapy, so the TERRA level is comparatively higher in P9 than in P7/P10. On the contrary, the other patients who did not receive combined therapy had much higher TERRA levels. We do not know whether a low TERRA level is associated with the responsiveness of AA patients against combined androgen with prednisolone therapy. Anyway, the strength of this work is that our data regarding telomere length, telomerase activity, and deregulated expression of telomere-associated proteins are corroborated with other scientific reports as described above. The novelty of this work is that this is going to be the first report on the association of telomeric non-coding RNA TERRA expression with aplastic anemia. However, we studied with a limited number of patients (n=11). To confirm this, we need to study a larger number of patients. Combined therapy induces regeneration of new blood cells, which is most likely contributing here and showing a low TERRA level. Our data indicates a significantly higher expression of TERRA and TIN2 in the blood cells of AA patients. Further research with a larger patient cohort is needed to determine whether TERRA or TIN2 could serve as reliable biomarkers for AA.

## Conclusions

Our findings highlight common features among aplastic anemia patients, along with the molecular parameters such as shortened telomeres, reduced telomerase activity due to significant low expression of hTERT and hTERC, and altered expression of telomere-associated proteins. Elevated TERRA and TIN2 levels may serve as indicators of aplastic anemia in patients under 12 years old.
